# Bridging the health inequality gap: an examination of South Africa’s social innovation in health landscape

**DOI:** 10.1186/s40249-021-00804-9

**Published:** 2021-03-01

**Authors:** Katusha de Villiers

**Affiliations:** Bertha Centre for Social Innovation and Entrepreneurship, The University of Cape Town Graduate School of Business, Cape Town, South Africa

**Keywords:** South Africa, Acceptability, Accessibility, Affordability, Health, Equity, Social innovation

## Abstract

**Background:**

Despite the end of apartheid in the early 1990s, South Africa remains racially and economically segregated. The country is beset by persistent social inequality, poverty, unemployment, a heavy burden of disease and the inequitable quality of healthcare service provision. The South African health system is currently engaged in the complex project of establishing universal health coverage that ensures the system’s ability to deliver comprehensive care that is accessible, affordable and acceptable to patients and families, while acknowledging the significant pressures to which the system is subject. Within this framework, the Bertha Centre for Social Innovation & Entrepreneurship works to pursue social impact towards social justice in Africa with a systems lens on social innovation within innovative finance, health, education and youth development. The aim of this study is to demonstrate the capacity for social innovation in health with respect for South Africa, and to highlight some current innovations that respond to issues of health equity such as accessibility, affordability, and acceptability.

**Methods:**

Different data types were collected to gain a rich understanding of the current context of social innovation in health within South Africa, supported by mini-case studies and examples from across the African continent, including: primary interviews, literature reviews, and organisational documentation reviews. Key stakeholders were identified, to provide the authors with an understanding of the context in which the innovations have been developed and implemented as well as the enablers and constraints. Stakeholders includes senior level managers, frontline health workers, Ministry of Health officials, and beneficiaries. A descriptive analysis strategy was adopted.

**Results:**

South Africa’s health care system may be viewed, to a large extent, as a reflection of the issues facing other Southern African countries with a similar disease burden, lack of systemic infrastructure and cohesiveness, and societal inequalities. The evolving health landscape in South Africa and the reforms being undertaken to prepare for a National Healthcare Insurance presents the opportunity to understand effective models of care provision as developed in other African contexts, and to translate these models as appropriate to the South African environment.

**Conclusions:**

After examining the cases of heath innovation, it is clear that no one actor, no matter how innovative, can change the system alone. The interaction and collaboration between the government and non-state actors is critical for an integrated and effective delivery system for both health and social care.
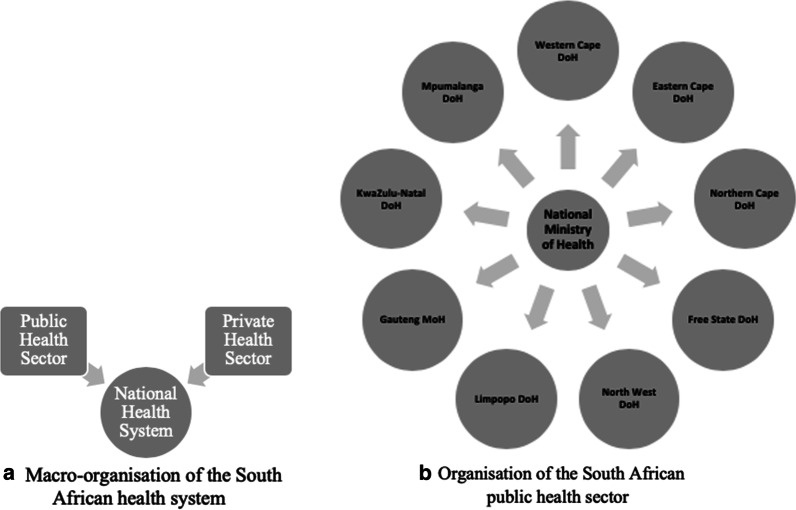

## Background

South Africa is a land of painful contrasts. Its vast mountain ranges, rolling farmland and vineyards, and glittering cities overlook the reality of informal settlements, unreliable and unsafe transportation routes, and rampant unemployment. This is certainly not what was envisioned when the work began of dismantling apartheid, a system of institutionalized racial segregation that had existed in the country since 1948. Following the release of Nelson Mandela in February 1990, after more than 27 years of incarceration, the South African government, the African National Congress, and various other political organizations embarked on a series of negotiations that peacefully brought about the end of the apartheid regime. These negotiations culminated in South Africa’s first non-racial elections in 1994, which were supposed to usher in a new era where all South Africans, in the words of Nelson Mandela, could embrace the “cherished … ideal of a democratic and free society in which all persons live together in harmony and with equal opportunities.”

Despite this early promise, South Africa remains racially and economically segregated. The country is beset by persistent social inequality, poverty, unemployment, a heavy burden of disease and the inequitable quality of health service provision. Challenges within the social care environment in South Africa include lack of access to the basic requirements of life such as clean water, nutrition, sanitation and education. The country has seen substantial economic growth in the post-apartheid era, yet over half of South Africa’s population lives in poverty [1]. The disparity between socio-economic groups within the country is one of the widest in the world; in 2018, the World Bank released a report on poverty and inequality in South Africa, and named it the most inequal society globally due not only to income inequality but also to wealth inequality. In its report, the World Bank found that the top one percent of South Africans control 70.9% of the country’s wealth while 60% of the country’s population collectively controls only seven percent of the country’s assets [2]. Despite the end of apartheid, this split is largely still along racial lines.

Post-apartheid, the South African government has invested heavily in social programs with the goal of reducing poverty and inequality. These include the establishment of the Black Economic Empowerment program, geared towards investment in black owned businesses; the expansion of safety net schemes such as free primary education, the development of a National Health Insurance plan, and promotion of minimum income grants to parents; and ambitious infrastructure projects improving access to water, sanitation, housing and healthcare facilities. In many ways, the South African government’s social protection program is efficient and efficacious, but in its totality, its degree of success is sporadic. Poverty levels in the country have been growing since 2015, and those at greatest risk include children, black Africans, people living in rural and remote areas, and those who have little or no access to education [1]. In 2015, more than 30 million South Africans, or nearly 56% of the population, lived on less than United States Dollolar (USD) 5 per day; this is still true in 2019 [2].

Arguably, a rationale for these sobering statistics is the simple fact that South Africa’s legacy of exclusion means that the country’s white minority, some nine percent of the population, still benefits from apartheid’s unequal policies. Those who have always had access to opportunity continue to have access—through intergenerational wealth and mobility, land ownership, quality education, and so on. This means that inequality is being passed down, and large numbers of South Africans remain vulnerable to the government’s inability to address this extreme disparity.

Within this framework, the Bertha Centre for Social Innovation & Entrepreneurship, a specialized unit within the University of Cape Town’s Graduate School of Business, works to pursue social impact towards social justice in Africa, through teaching, knowledge-building, convening and catalytic project work with a systems lens on social innovation within innovative finance, health, education and youth development. Within the context of the Bertha Centre’s work and activities, social innovation is defined as “A novel solution to a social problem that is more effective, efficient, sustainable, or just than existing solutions and for which the value created accrues primarily to society as a whole rather than private individuals” [3].

At the Bertha Centre, we believe that through inclusivity and radical collaboration, social innovation in health can open the doors to broadscale systemic impact by bringing together different disciplines, emphasizing co-creation, and pioneering solutions and business models that respond to real health needs. Through exploring new ways of doing, being and understanding, we hope to achieve a more equal society which works towards overcoming and addressing social and environmental challenges [4].

## Methods

A few examples of innovative health solutions within South Africa and elsewhere in Africa are described below and were investigated by the Bertha Centre through desk research, site visits, and numerous interviews with founders, staff, funders, and government officials. These cases were selected for inclusion based on the definition of social innovation included earlier in this writing.

## Discussion and results

### The health system in South Africa

The South African healthcare system is currently engaged in the complex project of establishing universal health coverage that ensures the system’s ability to deliver comprehensive care that is accessible, affordable and acceptable to patients and families, while acknowledging the significant pressures to which the system is subject. South Africa faces healthcare challenges in three major areas: the growing quadruple disease burden (see below); systemic and structural challenges in service delivery; and societal challenges associated with poverty and unemployment.

The vast burden of diseases is a major challenge, and the overburdened public health system is unable to accommodate increased demand. South Africa’s quadruple burden of diseases consists of HIV and AIDS, communicable disease, non-communicable disease, and violence and injuries. The consequence of these colliding epidemics is high levels of mortality and morbidity [5]. South Africa has the world’s largest population of people living with HIV—7.1 million people are afflicted—and one of the highest incidence rates of multi-drug resistant tuberculosis in the world [6]. South Africa is further experiencing a growing burden of chronic non-communicable disease. In 2016, cardiometabolic conditions (diabetes, cerebrovascular, heart and hypertensive disease) and other non-communicable diseases accounted for 57.4% of deaths in the country [7].

The South African health system faces a range of systemic and structural challenges, which include widespread inefficiencies, staff shortages, variability in skill sets between rural and urban areas, and suboptimal care levels and patient management [8]. The healthcare system consists of a large poorly funded public sector and a smaller better resourced private sector. The national Department of Health holds overall responsibility for healthcare, and specifically for the public sector across South Africa’s nine provinces. Nearly half of public health resources are allocated to district health services, which include primary healthcare clinics, community health centres and district hospitals.

Healthcare remains the load of the state because of high levels of poverty and unemployment, and the persistent inequality between public and private sector healthcare has created a system in which the public sector is overburdened in comparison to the private sector. The public sector provides services to roughly 84% of the population without private health insurance, and yet the government spends less than half the total health expenditure on healthcare [9]. Annual per capita expenditure on health ranges from USD 1400 per patient in the private sector to approximately USD 140 in the public sector. This discrepancy is also reflected in the availability of healthcare providers across the two spheres. Despite being one of the top five African countries in terms of density of medical personnel per 1000 population, the South African public health system is chronically understaffed, particularly in rural and remote areas [10]. Around 70% of doctors work only in the private sector, which leaves 30% of physicians available to service the public sector [9].

At the time of this writing, South Africa is introducing universal health coverage, under the banner of a National Health Insurance (NHI) system, that is envisaged as a response and solution to these three major challenges. The goal of the NHI is to foster healthcare reform to improve service provision and healthcare delivery for all socioeconomic groups, while also partnering with providers and organizations within the private sector in the delivery of healthcare. Private sector healthcare and medical aid schemes will remain in existence, but the NHI is intended to ensure that South Africans are able to access both the public and the private sectors through a blended model that will provide services in a manner that will benefit the entire population. In 2012, the NHI began a phasing process that will see it into existence over a period of 14 years. However, the implementation of the NHI only serves to accentuate the scale of the interventions necessary to ensure a well-functioning health system. The public sector’s critical shortages, maldistribution of resources, and underrepresentation of medical personnel and other health professionals highlights the urgent need for a more integrated health system, and the necessity of improving quality of care, access to services and general health equity [11].

In addition, apartheid-era urban planning means that public services, institutions and facilities remain inaccessible and inconvenient to large numbers of South Africans. Cape Town’s Groote Schuur Hospital serves as a pertinent example. A large, government-funded, tertiary and quaternary care facility, Groote Schuur functions as the University of Cape Town’s teaching hospital. It is located in Cape Town’s Observatory suburb, a far distance for patients who have been referred for care from the townships on the city’s periphery. Transport into the Cape Town city center is expensive, unreliable and unsafe. A commute for a hospital visit could cost up to USD 3, when an average service-sector job pays as little as USD 10 per day [12].

In the context of deep inequality, new methods of addressing and overcoming social and environmental challenges need to be explored. Due to its status as a developing country and emerging economy, South Africa is a ripe testing ground for entrepreneurship and social innovation. Entrepreneurs and innovators do not feel bound by traditional solutions and are able to reach members of the country’s most vulnerable populations. In addition, the sheer magnitude of the health challenge has led to the realisation that improving health outcomes will require the collaboration of the public and private sectors, as well as civil society. This realisation echoes the findings of Mason et al. in their determination that “a prevailing characteristic of social innovation is its responsiveness to failures or shocks of economic, social welfare, and wider political systems” [13] and that its function can be interpreted as a response to “failures of or gaps in institutional systems” [13].

Healthcare, and services promoting health as a resource, in South Africa, and across the continent, is ripe for systemic innovation that capitalizes on resource scarcity, because it allows for new methods and technologies to be adopted more quickly. These innovations can take many forms, for example, where mobile technology is used to provide health services and information, risk pooling where micro-insurance providers can tailor products to lower-income markets, and service provision, where services are re-engineered to achieve quality yet affordable healthcare [14]. The work of social innovation provides an opportunity to develop transformative and systemic solutions that move the system as a whole closer to achieving health equity, embodying what Mason et al. terms its greatest value i.e. “its capacity to redress system failures at local levels” [13].

### Access to health and case studies

Although government remains a key provider of basic services, social innovations are primarily developed at the frontlines of healthcare delivery by individuals and communities in response to a pressing need, often not met by government services. These social innovations take various forms from technological products, processes, novel organisational models or market mechanisms. Social innovation is most often implemented and tested at a local, grass-roots level before being extended to district, provincial, and national levels, and is often adopted at an ad-hoc fashion. A few examples of innovative health solutions within South Africa and elsewhere in Africa are described below.

The World Health Organization defines equity and health equity as follows:

Equity is the absence of avoidable, unfair, or remediable differences among groups of people, whether those groups are defined socially, economically, demographically or geographically or by other means of stratification. "Health equity” or “equity in health” implies that ideally everyone should have a fair opportunity to attain their full health potential and that no one should be disadvantaged from achieving this potential [15]. The simplistic argument could be that simple access to health is ipso facto health equity. However, a better way to appreciate the dilemma is to consider access to health along three dimensions, and to posit that if a health system were to view these dimensions as a lens through which it understands and responds to its challenges, it might move closer to achieving a greater degree of health equity.

### Physical accessibility

The first dimension of access to health is physical accessibility. This speaks to the availability of good health services within reasonable reach of those who need them and of opening hours, appointment systems and other aspects of service organization that allow people to obtain the services when they need them.

Imagine you have a 2-year-old who wakes up one day with a fever and you realise that she could have malaria. You know that the only way to get her the medicine, and you need to walk or hitchhike 50 km to reach the nearest clinic. This is the reality for many parents who live in rural and remote areas of South Africa. Health innovation is bridging this gap between the patient and the healthcare facility. An example is the Transnet Phelophepa Health Trains, which use South Africa’s railway network to take mobile clinics into the country’s heartland, treating 200 000 patients per year [16].

Imagine that you have a chronic illness, but your work requires you to travel from your township home on the urban periphery into the city centre, a journey that could take you up to 3 h. Where would you find the time to visit a health facility within its opening hours to pick up your chronic medication? Sizwe Nzima developed a solution that addresses these issues of accessibility and convenience that face many people in Cape Town’s poorer areas. In 2013, he established Iyeza Express, a bicycle courier service employing local youth as specialised medical couriers. His team collects chronic medications from public health facilities and delivers them directly to people’s homes, providing valuable services to over 1000 people living in Khayelitsha township. Iyeza Express has grown into Iyeza Health, a health logistics company that aims to strengthen public health systems through improved access to community-based patients.

Similar examples can be found across Africa. VillageReach in Malawi has developed a proactive model that works to create an enabling context and connect key players — including cellphone service providers — to enable healthcare delivery in the last mile, or that final link between a patient and the healthcare system. *Chipatala cha pa foni* (meaning Health Center by Phone), its toll-free health hotline, provides community members in remote areas with the opportunity to interact with the health system without having to travel long distances to the nearest health facility. The Ihangane Project in Rwanda provides another example; by engaging with health workers and patients, the organisation works to build community ownership of local health systems. Since 2016, it has worked with communities across Rwanda’s Ruli district to build eight community health buildings which are used to provide village-level access to health care, while the buildings also serve as locations for education and community meetings.

### Financial affordability

The second dimension of access to health is financial affordability. This is the measure of a person’s ability to pay for services without financial hardship. It takes into account not only the price of the health services but also indirect and opportunity costs (e.g. the costs of transportation to and from facilities and of taking time away from work). Affordability is influenced by the wider health financing system and by household income.

In 2013, entrepreneur Neo Hutiri was diagnosed with tuberculosis. During his 6-month treatment period, he had to travel to the Bophelong health clinic in Johanesburg every 2 weeks, where he would queue for hours to pick up his medication. During that period, he had plenty of time to think: what if he was able to create a solution that would allow patients to collect their repeat medications in a matter of minutes rather than hours? The Pelebox Smart Locker is such a solution. Instead of incurring the indirect and opportunity costs of queuing at the health clinic, patients simply receive a one-time PIN on their phone, which they use to unlock the locker to retrieve their medication [17]. Neo’s solution has the potential to improve a healthcare system under great strain, and this has been recognised; Neo has partnered with the Department of Health since 2016, and to date the Pelebox Smart Locker has allowed more than 8000 patient collections across Gauteng Province [18].

South Africa runs the largest HIV antiretroviral therapy programme in the world, and the bulk of those patients receive treatment in the public sector. HIV-positive patients require regular check-ups, adherence support and counselling services; however, their compliance with their treatment is compromised when the overburdened public health system does little to protect patient privacy [19]. The complex health challenge of HIV cannot be solved only within the public sector; it requires the collaboration of the public, private and not-for-profit sectors. Public-private partnerships (PPP) is one such collaboration: this is a mechanism through which private sector involvement in developing, financing and providing healthcare infrastructure and service delivery within the public sector is harnessed. One such PPP is the GP-Down Referral Model, launched by BroadReach Healthcare in 2005 in partnership with the North West Province Department of Health. The GP-Down model works by shifting the delivery of antiretroviral therapy of stable patients from the overburdened public sector to private independent general practitioners. The model frees up resources in the public sector, and improves the care offered to patients — now, they are able to consistently see the same physician — while also reducing the direct and indirect costs of having to access the public health system [19]. Through PEPFAR grants and subsidies in kind from the provincial government, this co-funded model allows BroadReach to pay for model set-up and staffing costs, as well as providing patient adherence support and covering the costs of GP consultation fees; in its turn, the North West Province Department of Health provides the antiretrovirals as well as the laboratory support [19]. Patients reported that they valued the chance to build more trusting relationships with their doctors, while they were also able to save money and visit a clinic closer to home where appointments were timely.

### Acceptability

The third and final dimension of access to health is acceptability. This captures a person’s willingness to seek services. Acceptability is low when patients perceive services to be ineffective or when social and cultural factors such as language, age, sex, ethnicity or religion of health provider discourage them from seeking services.

The issues of language and culture in South Africa are applicable examples of how important acceptability is in seeking out healthcare services, and in truly being able to access health as a resource. South Africa recognizes eleven official languages, of which English is the primary language for state discourse, and more often than not, the primary language of the health community. This can be very isolating to patients whose mother tongue is isiXhosa, Venda, or Zulu, and who, in consequence, cannot communicate with their healthcare provider or might need to rely on a child or a nurse as a translator. This is the case for many patients who live in the Umkhanyakude district of KwaZulu-Natal. There is little electricity, running water or sanitation in this region, and its population of more than half-a-million is served by only five public hospitals which are beset by chronic shortages of professional healthcare staff [16]. In response, The Umthombo Youth Development Foundation offers an innovative solution by addressing the skills shortages in rural health by providing scholarships for promising young people to study, who then return to their home communities as doctors, nurses, social workers, physiotherapists, and other healthcare professionals. These graduates are able to communicate with their patients in their own language, and are held in high esteem by their communities [16]. At the time of this writing, the Foundation has supported 385 graduates; of those graduates, 177 no longer have further work back obligations. Of these 177 healthcare professionals, 65% still work in rural facilities and an additional four percent are working with rural NGO’s [20].

An example beyond South Africa is Last Mile Health, a health innovator located in Liberia. In partnership with the Liberian government, Last Mile Health trains community health workers to prevent, diagnose and treat a range of medical conditions and diseases using smartphone technology. These community health workers address the needs of people in remote communities that lack access to care due to distance and poverty. By recruiting, training, equipping and paying community members to deliver lifesaving health services to their neighbours, Last Mile Health ensures that patients are able to access care from a trusted healthcare professional who they know, understand, and believe will provide them with the appropriate treatment and medication for common medical conditions such as malaria and pneumonia.

## Conclusions

South Africa’s health care system may be viewed, to a large extent, as a reflection of the issues facing other Southern African countries with a similar disease burden, lack of systemic infrastructure and cohesiveness, and societal inequalities. And yet the evolving health landscape in South Africa and the reforms being undertaken to prepare for a National Healthcare Insurance presents the opportunity to understand effective models of care provision as developed in other African contexts, and to translate these models as appropriate to the South African environment. A main focus of the South African Ministry of Health in regard to the National Health Insurance is the delivery of high-quality accessible primary care to all citizens and, thus, research on translational models of primary care hold value for local policy makers. Kenya’s innovative approach to primary health care provision is particularly ripe for examination, as, contrary to South Africa, almost half of Kenya’s poor utilizes services provided by the private health sector. South Africa could also gain relative to its policy and regulatory environment and the innovation ecosystem of other African countries. For example, mobile health has been used more extensively in countries such as Kenya and Uganda, as compared to South Africa, to support care delivery in low-income communities.

No one actor, no matter how innovative, can change the system alone. The interaction and collaboration between the government and non-state actors is critical for an integrated and effective delivery system for both health and social care. The social innovation felt at a grass-roots level must be celebrated and promoted on a national level so that successful ideas, products, services and solutions are connected to policy makers at all levels of government. Across the health system and within communities, there are individuals with the potential of making a valuable contribution that could achieve positive systemic change. The value and importance of each of these change agents need to be recognized, nurtured and supported such that a broader collective agenda of equity, access and accountability can be achieved.

South Africa can be viewed as a microcosm of existing global challenges—inequality, youth unemployment, poverty, health inequity. These challenges can seem overwhelming, but South Africa has a strong infrastructure, media freedom, an active civil society, a relatively robust economy, and most importantly, amazing human talent and possibility [21]. As a result, the country has the right active ingredients to make real progress, and so contribute to providing African solutions to African problems.

## Data Availability

Not applicable.

## Abbreviations

NHI: National Health Insurance
PPP: Public Private Partnership
PEPFAR: The President’s Emergency Plan for AIDS Relief
NGO: Non-Governmental Organisation
USD: United States Dollar
